# Prognostic impact of catheter ablation in atrial fibrillation with heart failure with preserved ejection fraction: an updated systematic review and reconstructed individual patient data meta-analysis

**DOI:** 10.3389/fcvm.2026.1883350

**Published:** 2026-08-19

**Authors:** Srilakshmi Ajith, Marc Knezevic-Maragh, Daniel Bradach, Basel F. Alqeeq, Mohammed Al-Tawil, Assad Haneya

**Affiliations:** 1 University Hospitals Birmingham, Birmingham, United Kingdom; 2 Ross University School of Medicine, Bridgetown, Barbados; 3 St.George’s University School of Medicine, University Centre Grenada, St. George's, Grenada; 4 Cardiovascular Research Institute (CVRI), Mater Private Network, Dublin, Ireland; 5 Department of Cardiac and Thoracic Surgery, Trier Heart Centre, Trier, Germany

**Keywords:** atrial fibrillation, catheter ablation, heart failure with preserved ejection fraction, hospitalization, meta-analysis, mortality, reconstructed individual patient data

## Abstract

**Background:**

Catheter ablation improves outcomes in atrial fibrillation (AF) with heart failure with reduced ejection fraction, but its efficacy in heart failure with preserved ejection fraction (HFpEF) remains uncertain. We performed a systematic review and reconstructed individual patient data meta-analysis to evaluate catheter ablation compared with medical therapy in this population.

**Methods:**

Electronic databases were searched from inception through December 8, 2025, for randomized and observational studies comparing catheter ablation with medical therapy in adults with AF and HFpEF (LVEF ≥50% or guideline-consistent definitions). Primary outcomes were all-cause mortality and heart failure (HF) hospitalizations.

**Results:**

Across 13 studies (45,642 patients), catheter ablation was associated with significantly lower all-cause mortality (HR 0.55, 95% CI 0.42–0.73; *p* < 0.0001), reduced HF hospitalizations (HR 0.67, 95% CI 0.52–0.86; *p* = 0.001), lower stroke/TIA risk (RR 0.89, 95% CI 0.79–0.99; *p* = 0.04) and reduced AF/atrial arrhythmia recurrence (HR 0.59, 95% CI 0.51–0.69; *p* < 0.00001). Reconstructed Kaplan–Meier curves demonstrated consistent time-to-event separation favouring catheter ablation across these outcomes. All-cause hospitalizations were lower (HR 0.90, 95% CI 0.81–1.00; *p* = 0.05), but not significant. Exploratory analyses demonstrated directionally favourable reductions in NT-proBNP and small changes in LVEF following ablation.

**Conclusion:**

Catheter ablation may confer prognostic benefit in selected AF and HFpEF populations, though large, randomized trials are needed to confirm these findings.

**Systematic Review Registration:**

https://www.crd.york.ac.uk/PROSPERO/view/CRD420261278738, PROSPERO, CRD420261278738.

## 1 Introduction

The coexistence of atrial fibrillation (AF) and heart failure with preserved ejection fraction (HFpEF) is prevalent and significantly contributes to morbidity and mortality (1). The public health burden associated with both conditions is increasing, with projections indicating that by 2030, approximately 12 million individuals in the United States will have AF and more than 8 million will have heart failure (2, 3).

The relationship between AF and heart failure (HF) is both bidirectional and multifactorial. AF may facilitate the development and progression of heart failure through mechanisms such as loss of atrial systole, irregular ventricular rate, tachycardia, neurohumoral activation, and adverse myocardial remodeling. Conversely, structural atrial remodeling, mitral regurgitation, and neurohumoral dysregulation associated with heart failure increase susceptibility to AF. Both conditions share cardiovascular risk factors, including advanced age, hypertension, diabetes, obesity, smoking, and obstructive sleep apnea (4).

Catheter ablation of AF has demonstrated improved outcomes in patients with heart failure with reduced ejection fraction (HFrEF), and current international guidelines recommend AF ablation for selected patients with left ventricular systolic dysfunction (5). However, the efficacy of catheter ablation in HFpEF remains uncertain, as previous meta-analyses have reported conflicting results (6, 7).

Previous meta-analyses have been limited by heterogeneous definitions of heart failure and the inclusion of overlapping HFmrEF and HFrEF populations (8, 9). As more recent studies report outcomes in well-defined HFpEF cohorts, a reassessment of the evidence is warranted. This systematic review and meta-analysis therefore aims to evaluate the efficacy of catheter ablation compared with medical therapy in patients with HFpEF, prioritizing studies with clearly defined HFpEF populations or extractable HFpEF-specific subgroup data.

## 2 Methods

### 2.1 Protocol, reporting standards, and registration

This systematic review and meta-analysis was performed according to the Preferred Reporting Items for Systematic Reviews and Meta-analyses (PRISMA) guidelines. The research question and eligibility framework were prespecified using the PICOTT approach, focusing on adults with AF and HFpEF undergoing catheter ablation compared with medical therapy/no ablation, with mortality and HF hospitalization as primary outcomes. The study was registered on the International Prospective Register of Systematic Reviews (PROSPERO), CRD420261278738.

### 2.2 Search strategy and information sources

A comprehensive electronic literature search was conducted in PubMed, Embase, and Scopus from inception to December 8, 2025. The core search strategy included terms for “ablation”/ “catheter ablation,” “atrial fibrillation,”, “HFpEF/heart failure preserved ejection fraction” and “heart failure”. Reference lists of included studies and relevant reviews were manually screened, and citation searching was performed to identify additional eligible studies.

### 2.3 Eligibility criteria

Studies were eligible if they included enrolled adults aged ≥18 years with concomitant AF and HFpEF, compared catheter ablation with medical therapy and/or no ablation, were randomized controlled trials (RCTs) or observational cohort studies, and reported at least one prespecified outcome. Given the variability in HFpEF definitions across different studies, HFpEF was defined as a left ventricular ejection fraction (LVEF) ≥ 50% or according to study-specific definitions consistent with contemporary guideline-based criteria. Studies including mixed heart failure populations were eligible only if HFpEF-specific subgroup data were extractable.

Exclusion criteria included review articles, case reports/series, non-original articles, conference abstracts, non-English publications, pediatric studies, studies without a comparator group, and studies focused on HFrEF/HFmEF, acute decompensated heart failure, or cardiogenic shock without separable HFpEF data.

### 2.4 Study selection

All records were imported into Rayyan, a reference management software, and deduplicated. Titles/abstracts were screened independently by two reviewers, followed by full-text review of potentially eligible articles. Disagreements were resolved through discussion or adjudication by a third reviewer.

### 2.5 Data extraction and outcomes

Data extraction was performed independently using a standardized form. Variables collected included study characteristics, HFpEF definition, AF characterization, ablation and comparator strategies, sample size, follow-up duration, adjustment methods, and outcome data.

The primary outcomes were all-cause mortality and heart failure hospitalizations. Secondary outcomes included stroke or TIA, AF or atrial arrhythmia recurrence, all-cause hospitalization, and changes in N-terminal pro-B-type natriuretic peptide (NT-proBNP) and LVEF.

AF or atrial arrhythmia recurrence was defined as rhythm-documented recurrence on ECG or device-based monitoring. Studies that reported AF-related hospitalization without rhythm confirmation were excluded to minimize endpoint misclassification.

When multiple adjusted estimates were available, the most fully adjusted estimate, such as those using propensity-score adjustment or multivariable models, was selected. For studies reporting outcomes at multiple time points, the longest comparable follow-up period was used for data pooling.

### 2.6 Risk of bias assessment

Risk of bias was assessed independently by two reviewers. Observational cohort studies were evaluated using the Newcastle–Ottawa Scale (NOS), and RCTs were evaluated using the Cochrane Risk of Bias tool (RoB 2). Disagreements were resolved through discussion and consensus.

### 2.7 Statistical analysis

Meta-analyses were conducted using Cochrane RevMan software, employing a random-effects model to address both clinical and methodological heterogeneity.

Time-to-event outcomes were pooled as hazard ratios (HRs) with 95% confidence intervals (CIs) using the inverse-variance method on log-transformed HRs. When HRs were not directly reported, log HRs and standard errors were reconstructed from Kaplan–Meier (KM) curves using the method described by Tierney et al. Survival curves were digitally extracted using validated graph digitization software, incorporating reported numbers at risk when available.

Dichotomous outcomes reported as event counts (e.g., stroke/TIA) were pooled as risk ratios (RRs) with 95% CIs using the Restricted Maximum-Likelihood method. Continuous outcomes were analyzed as mean change from baseline with corresponding standard deviations (SDs). When SDs of change were not reported, they were estimated using the correlation-based method recommended by the Cochrane Handbook (*r* = 0.7). Outcomes reported using non-comparable summary measures or with insufficient variance data were summarized narratively.

Statistical heterogeneity was assessed using Cochran's *Q* test and quantified with the I² statistic. A two-sided *p* value less than 0.05 was considered statistically significant. Sensitivity analyses included leave-one-out procedures to evaluate the influence of individual studies. Prespecified subgroup analyses were conducted based on median follow-up duration (greater than 2 years vs. 2 years or less), with subgroup differences assessed using Cochran's *Q* test.

Time-to-event data were further reconstructed from published Kaplan–Meier curves when sufficient graphical resolution and numbers at risk were available. Individual patient data (IPD) were reconstructed using the algorithm described by Liu et al., estimating event and censoring times from digitized survival curves and reported numbers at risk. Studies lacking published time-to-event curves were not eligible for IPD reconstruction but were included in the aggregate meta-analysis if adjusted effect estimates were available.

All reconstruction analyses were performed using R software (R version 4.5.1; R Foundation for Statistical Computing, Vienna, Austria). Survival analyses were conducted using the survival package with frailty-adjusted Cox proportional hazards models to account for between-study clustering. Kaplan–Meier curves were generated using the survminer and ggplot2 packages.

Formal assessment of publication bias using funnel plots or regression-based methods was not performed due to the limited number of studies included in each meta-analysis (<10 per outcome), which reduces the reliability of such tests.

## 3 Results

### 3.1 Literature search and included studies

**Figure 1:**
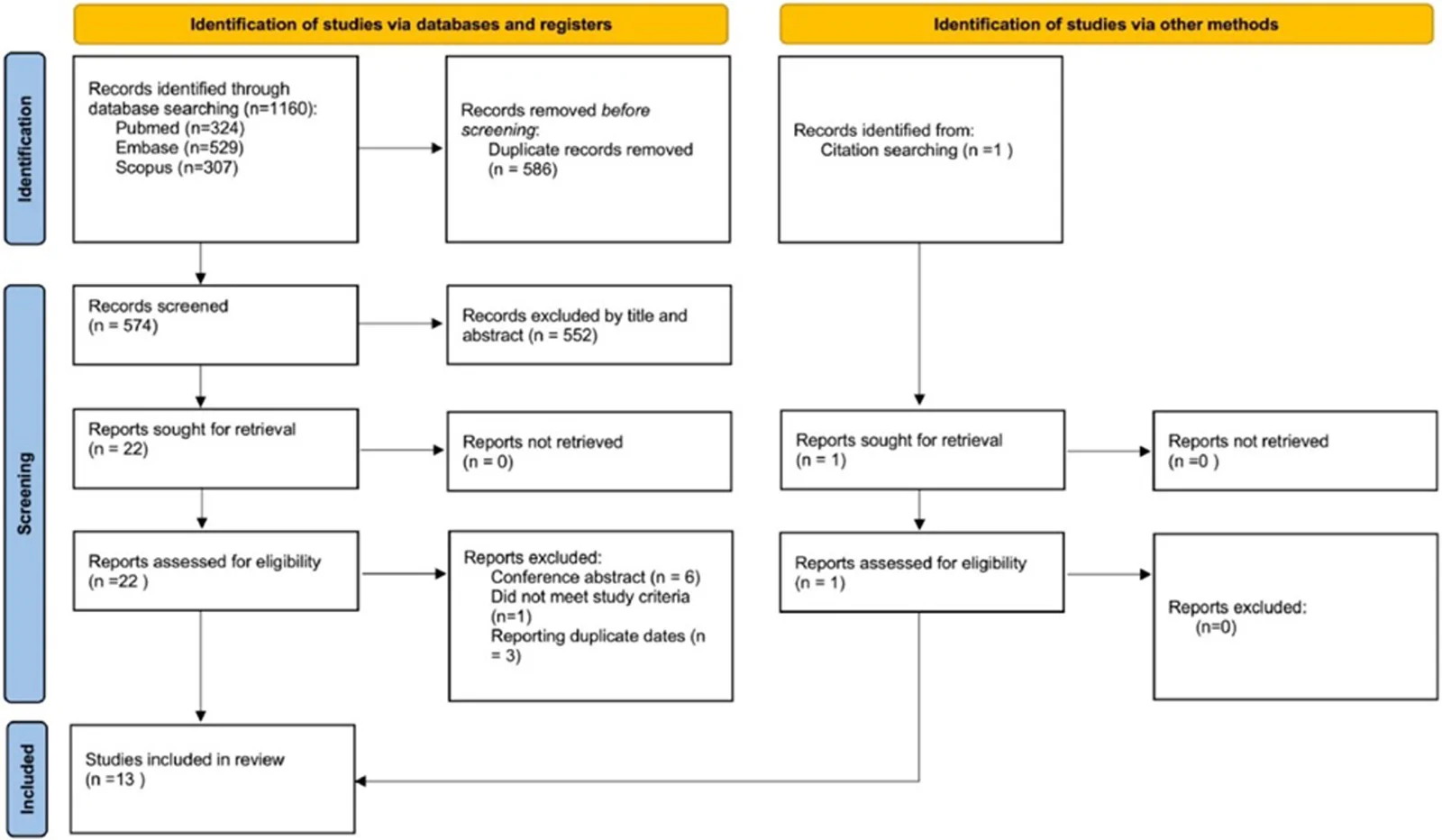
Preferred reporting items for systematic reviews and meta-analyses (PRISMA) flow diagram of study screening and selection.

A systematic literature search yielded 1,160 studies: 324 from PubMed, 529 from Embase, and 307 from Scopus. After removing 586 duplicates, 574 records were screened for eligibility based on titles and abstracts. Subsequently, 22 studies underwent full-text screening and citation chasing. Thirteen studies ultimately met the eligibility criteria and were included in both qualitative and quantitative syntheses. The study selection process was documented using a PRISMA flow diagram (Figure 1). A total of 45,642 patients undergoing catheter ablation vs. medical therapy were included in the analysis. The baseline demographic and clinical characteristics of included patients are summarized in Tables 1, 2. Detailed demographic data of included studies are provided in Supplementary Material S2 (Supplementary Table S1).

**Table 1:** Some included studies enrolled mixed heart failure populations. Only HFpEF-specific data were extracted when available. Follow-up duration is reported as provided in the original studies. For observational studies, adjusted or propensity score-matched cohorts were used when available. Administrative database studies defined AF and HFpEF using ICD/CPT codes. HFpEF definitions varied across studies and were based on study-specific criteria (e.g., LVEF "50%, guideline-based definitions, or probability-based scoring systems).

S. No	Study (Year)	Country	Data Source/Study Center	Study Period	Design	Patients (CA)	Patients (Med)	Follow-Up (CA)	Follow-Up (Med)	Key Inclusion Criteria	Key Exclusion Criteria	Main Findings/Conclusions	Notes
1	Arora et al., (10)	USA	NRD (HCUP)	2016–2017	Retrospective cohort	1,053	15,795	Median 184 days (≤1 yr)	Median 184 days (≤1 yr)	Adults with AF + HF (HFpEF/HFrEF via ICD-10)	CABG during index admission; missing data	No reduction in 1-yr mortality or HF readmission in HFpEF; AF readmissions reduced with CA	PSM cohort used; HFpEF subgroup data extracted
2	Chieng et al., (11)	Australia	Alfred & Royal Melbourne Hospitals	2018–2021	RCT (single-blind)	16	15	6 months	6 months	Symptomatic AF + invasively confirmed HFpEF	Long-standing AF, BMI >40, valvular disease	CA improved PCWP, peak VO₂, NT-proBNP, QoL; 50% no longer met HFpEF criteria	Mechanistic trial
3	DeLuca et al., (12)	USA	TriNetX	2017–2023	Retrospective cohort	1,816	1,816	≤6 years	≤6 years	HFpEF + AF on anticoagulation	Prior HFrEF, prior CA	Lower mortality and HF admissions with CA (HR 0.43 mortality)	Strong signal
4	Fukui et al., (13)	Japan	Oita University	2014–2018	Retrospective cohort	35	50	2 years	2 years	HFpEF with diastolic dysfunction	Age >85, cancer, ESRD	Reduced HF rehospitalization with CA; no AF recurrence difference	KM not statistically significant
5	Lee et al., (14)	Taiwan	Global TriNetX	2014–2024	Retrospective cohort	1,152	1,152	Median 773 days	Median 627 days	AF + HFpEF (EF ≥50%)	No anticoagulation, CKD/ESRD	Lower mortality, stroke, HF exacerbations with CA	Meets PICOTT
6	Long et al., (15)	China	Dalian Medical University	2019–2020	Retrospective cohort	187	187	36 ± 3 months	36 ± 3 months	Symptomatic AF + HFpEF (ASE/EACVI)	Devices, valvular disease	CA improved cardiac function; lower CV death, stroke, rehospitalization	—
7	Martens et al., (16)	USA	BioLINCC (CABANA)	2009–2016	Post-hoc RCT subgroup	488	486	∼48 months	∼48 months	AF with HFpEF probability score ≥6	Prior CA	CA reduced CV hospitalization/death; mortality alone unchanged	Probability-based HFpEF
8	Olshausen et al., (17)	Sweden	SwedeHF Registry	2005–2019	Retrospective cohort	72	151	Median 2.6 years	Median 2.6 years	AF + HFpEF (EF ≥50%)	Other atrial arrhythmias	CA associated with lower mortality & HF hospitalization	Small HFpEF subgroup
9	Parkash et al., (18)	Multinati onal	21 centers	2011–2018	RCT	214	197	Median 37 months	Median 37 months	AF + NYHA II–III HF	LA >55 mm, valvular disease	No reduction in mortality/HF events	Mixed HF cohort; HFpEF subgroup data extracted
10	Patel et al., (19)	USA	TriNetX	2010–2021	Retrospective cohort	9,440	9,440	24 months	24 months	AF + HFpEF via ICD/CPT	Age <18	Reduced mortality, HF readmission, stroke with CA	Large dataset
11	Rattka et al., (20)	Germany	Ulm University	2013–2018	Case-control	43	43	35 ± 22 months	35 ± 22 months	Symptomatic HFpEF + AF	Amyloidosis, shock	CA reduced AF recurrence, HF hospitalization; mortality similar	Detailed phenotyping
12	Xie et al., (21)	China	Multicenter	2015–2019	Retrospective cohort	293	293	Median 39 months	Median 39 months	HFpEF + AF, NYHA II–IV	Prior CA, ESRD	Lower mortality and thromboembolism with CA	—
13	Z. Le et al., (22)	China	Multicenter registry	2011–2020	Prospective cohort	604	604	4.2 years	4.2 years	AF + HF (HFpEF subgroup)	Age >80	CA reduced all-cause and CV mortality	Long follow-up

AF, atrial fibrillation; HF, heart failure; HFpEF, heart failure with preserved ejection fraction; HFrEF, heart failure with reduced ejection fraction; CA, catheter ablation; RCT, randomized controlled trial; NRD, Nationwide Readmissions Database; PSM, propensity score matched; EF, ejection fraction; NYHA, New York Heart Association; ESRD, end-stage renal disease; CKD, chronic kidney disease; PCWP, pulmonary capillary wedge pressure; QoL, quality of life; CV, cardiovascular; KM, Kaplan–Meier; HR, hazard ratio; NR, not reported.

**Table 2:** Baseline characteristics were summarized descriptively from reported study-level data. Not all studies reported every variable; therefore, denominators vary by characteristic.

Characteristic	Catheter Ablation	Medical/No Ablation
Number of patients (all included studies)	15,413	30,229
Age, years (mean ± SD)^a^	67.2 ± 9.7	66.6 ± 12.1
Male sex, *n* (%)^b^	7,401/13,440 (55.1%)	12,956/28,226 (45.9%)
Female sex, *n* (%)^b^	6,039/13,440 (44.9%)	15,270/28,226 (54.1%)
Hypertension, *n* (%)^b^	4,467/5,413 (82.5%)	17,221/20,152 (85.5%)
Coronary artery disease, *n* (%)^b^	859/2,509 (34.2%)	874/2,491 (35.1%)
Diabetes mellitus, *n* (%)^b^	1,657/4,360 (38.0%)	1,720/4,357 (39.5%)
Previous stroke/TIA, *n* (%)^b^	572/4,325 (13.2%)	599/4,307 (13.9%)
Persistent AF, *n* (%)^b^	697/1,111 (62.7%)	689/1,094 (63.0%)
Paroxysmal AF, *n* (%)^b^	126/507 (24.9%)	123/490 (25.1%)

AF, atrial fibrillation; HFpEF, heart failure with preserved ejection fraction; CA, catheter ablation; TIA, transient ischemic attack.

a Age pooled from studies reporting mean±SD

b Not all studies reported every variable; therefore, denominators vary by characteristic.

Figures depicting the Risk of Bias assessment are provided in the Supplementary Material S1 (Supplementary Figures S1, S2).

### 3.2 Primary outcomes

#### 3.2.1 All-cause mortality

**Figure 2:**
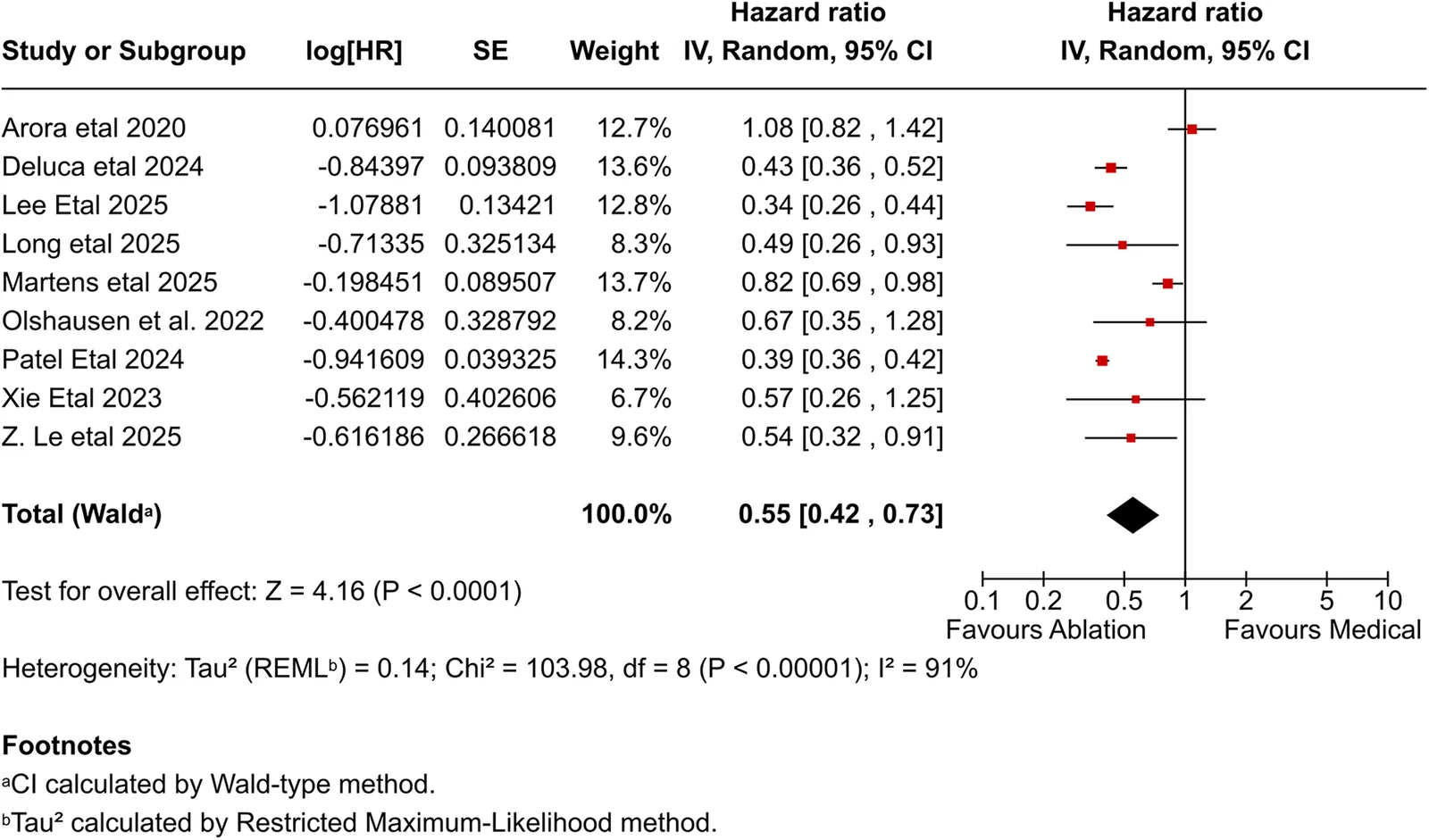
Forest plot showing All-cause mortality comparing catheter ablation vs medical therapy. Hazard ratios with 95% confidence intervals are shown. CI = Confidence interval; HR = hazard ratio; IV = inverse variance.

Based on random-effects meta-analysis conducted on nine studies, catheter ablation was associated with a significantly lower risk of all-cause mortality, compared to medical therapy (HR 0.55, CI 0.42–0.73, *p* < 0.0001) (Figure 2). Following a high heterogeneity, we performed a leave-one-out sensitivity analysis. Sequential exclusion of individual studies did not remarkably reduce heterogeneity. Prespecified subgroup analyses based on follow-up duration (median >2 years vs. ≤2 years) demonstrated that the association between catheter ablation and reduced all-cause mortality remained statistically significant in the subgroup with longer follow-up (>2 years), accompanied by a substantial reduction in heterogeneity (Supplementary Material S1, Supplementary Figure S3).

For the reconstructed time-to-event individual patient meta-analysis, 25,647 patients were analysed across treatment groups. The analysis showed that ablation was associated with a significant and marked survival benefit in patients with HFpEF. At 60 months, Kaplan–Meier estimates demonstrated higher survival in the ablation group compared with control (82.6% [95% CI 81.2–84.1] vs. 64.2% [95% CI 62.6–65.9]). In frailty-adjusted Cox proportional hazards modelling accounting for between-study heterogeneity, ablation significantly reduced mortality (HR: 0.384 [95% CI 0.359–0.410], *p* < 0.001) (Figure 6).

#### 3.2.2 HF hospitalizations

**Figure 3:**
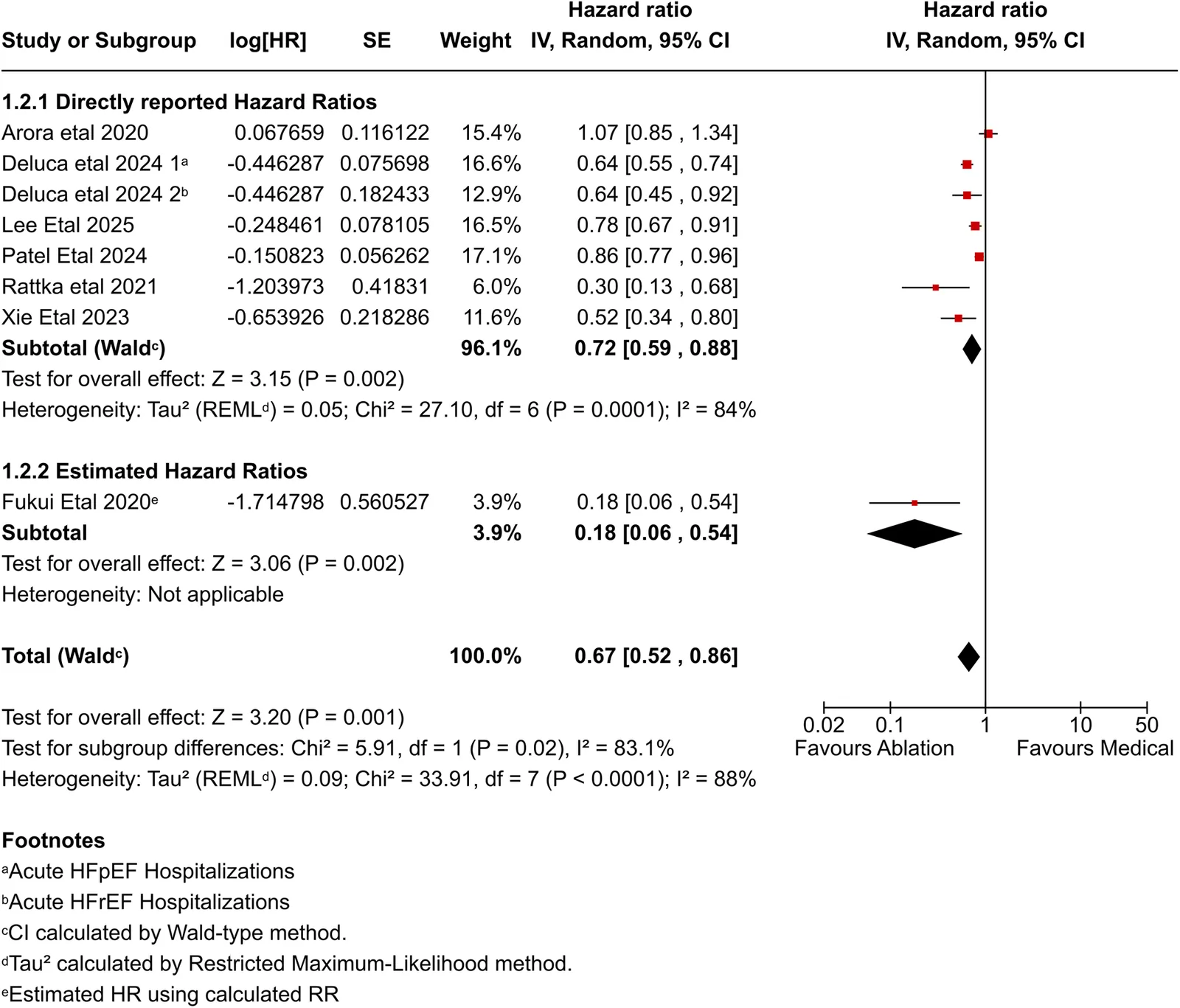
Forest plot showing heart failure hospitalization comparing catheter ablation vs medical therapy. Hazard ratios with 95% confidence intervals are shown. CI = Confidence interval; HR = hazard ratio; IV = inverse variance.

Seven eligible studies were synthesised using a random-effects model to evaluate for HF hospitalizations. Catheter ablation was associated with a significantly lower risk of HF hospitalizations compared to the medical group (HR 0.67, 95% CI 0.52–0.86, *p* = 0.001), with substantial heterogeneity (I^2^ = 88%) (Figure 3). Leave-one-out sensitivity analyses did not identify any single study whose exclusion materially reduced heterogeneity or altered the direction of effect. However, prespecified subgroup analysis by follow-up duration (>2 years vs. ≤ 2 years) demonstrated that the association remained significant in studies with longer follow-up (>2 years), with reduced heterogeneity (I^2^ = 50%) (Supplementary Material S1, Supplementary Figure S4).

In the study by DeLuca et al., heart failure hospitalizations were reported as two distinct HF-related outcomes, stratified by admission type (acute HFpEF admissions and acute HFrEF admissions) (12). As these outcomes were reported separately with distinct event counts within the HFpEF cohort, both estimates were entered independently into the meta-analysis and are presented as Deluca et al. (2024a) and (2024b) for transparency.

In reconstructed time-to-event analyses analyzing 6,242 participants, Kaplan–Meier estimates at 60 months demonstrated significantly higher freedom from HF readmission in the ablation group compared with control (54.4% [95% CI 52.2–56.6] vs. 39.5% [95% CI 37.7–41.3]). Frailty-adjusted modeling confirmed a significant reduction in the risk of HF readmission (HR: 0.635 [95% CI 0.591–0.683], *p* < 0.001) (Figure 6).

### 3.3 Secondary outcomes

#### 3.3.1 Stroke/TIA

**Figure 4:**
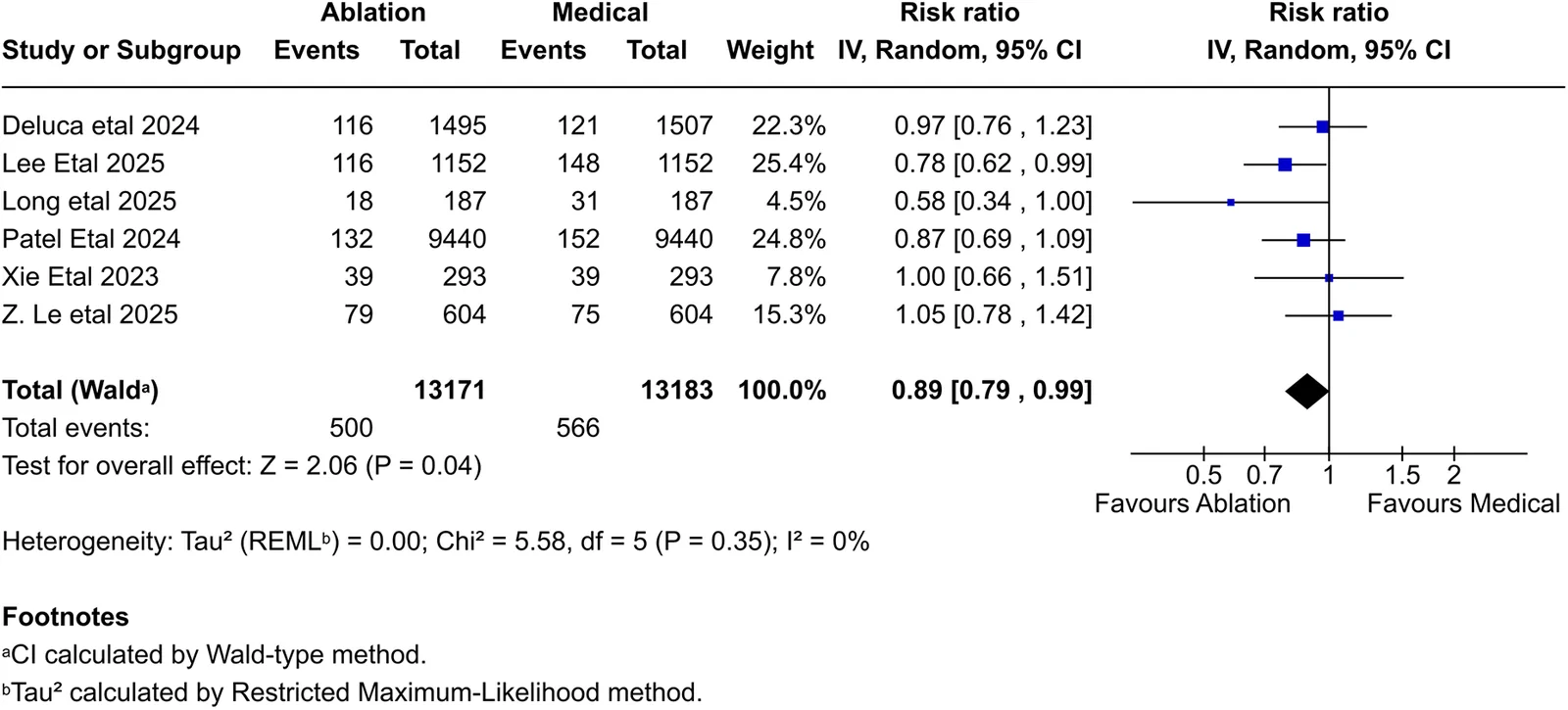
Forest plot showing stroke/TIA comparing catheter ablation vs medical therapy for. Risk ratios with 95% confidence intervals are shown. CI = Confidence interval; RR = Risk ratio.

Six studies reported this outcome (Figure 4). Catheter ablation was associated with a significantly lower risk of stroke/TIA compared with medical therapy (RR 0.89, 95% CI: 0.79–0.99; *p* = 0.04), corresponding to a 11% relative risk reduction. Heterogeneity among the studies was low (I^2^ = 0%), indicating consistent results across the studies. A sensitivity analysis restricted to studies reporting hazard ratios demonstrated a consistent reduction in stroke/TIA with catheter ablation (HR 0.74, 95% CI 0.62–0.89; *p* = 0.001), supporting the findings of the primary RR-based analysis (Supplementary Material S1, Supplementary Figure S5).

A total of 6,029 participants contributed to reconstructed time-to-event analyses. For stroke, 60-month Kaplan–Meier estimates demonstrated similar absolute freedom from stroke between groups, although numerically higher in the ablation arm (85.3% [95% CI 83.7–87.0] vs. 84.2% [95% CI 82.8–85.6]). In frailty-adjusted Cox modeling, ablation was associated with a significantly lower risk of stroke (HR: 0.684 [95% CI 0.593–0.791], *p* < 0.001), with a significant frailty term (variance 0.92), indicating between-study heterogeneity (Figure 6).

#### 3.3.2 AF/atrial arrhythmia recurrence

**Figure 5:**
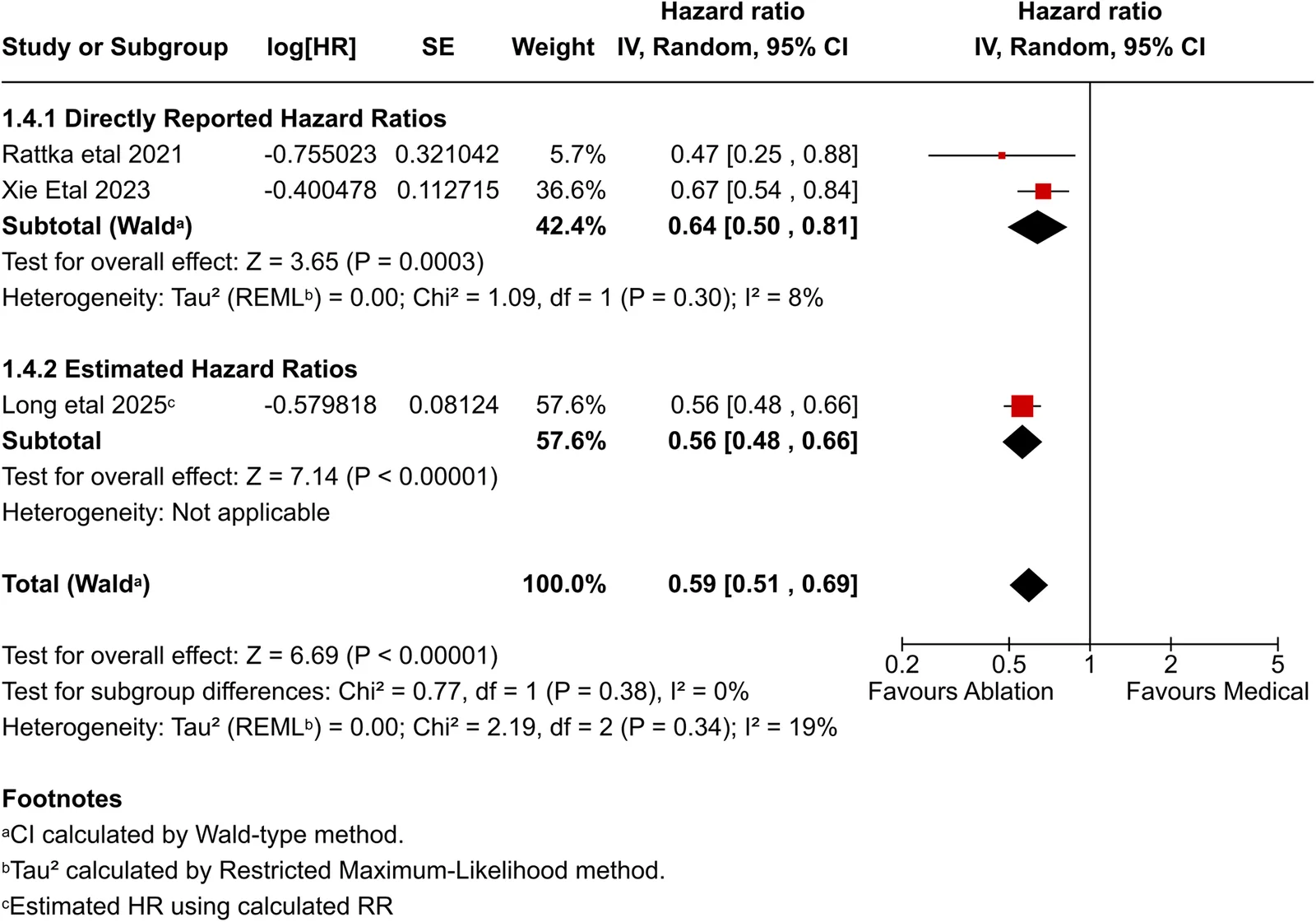
Forest plot showing atrial fibrillation/atrial arrhythmia recurrence comparing catheter ablation vs medical therapy. Hazard ratios with 95% confidence intervals are shown. CI = Confidence interval; HR = hazard ratio; IV = inverse variance.

AF/atrial arrhythmia recurrence was defined as rhythm-documented recurrence during follow-up. Three studies reported this outcome; two provided direct hazard ratios and one an estimated hazard ratio (Figure 5). Pooled analysis showed that catheter ablation was associated with a significantly lower risk of AF/atrial arrhythmia recurrence compared to medical therapy (HR 0.59, 95% CI 0.51–0.69; *p* < 0.00001; I^2^ = 19%).

**Figure 6:**
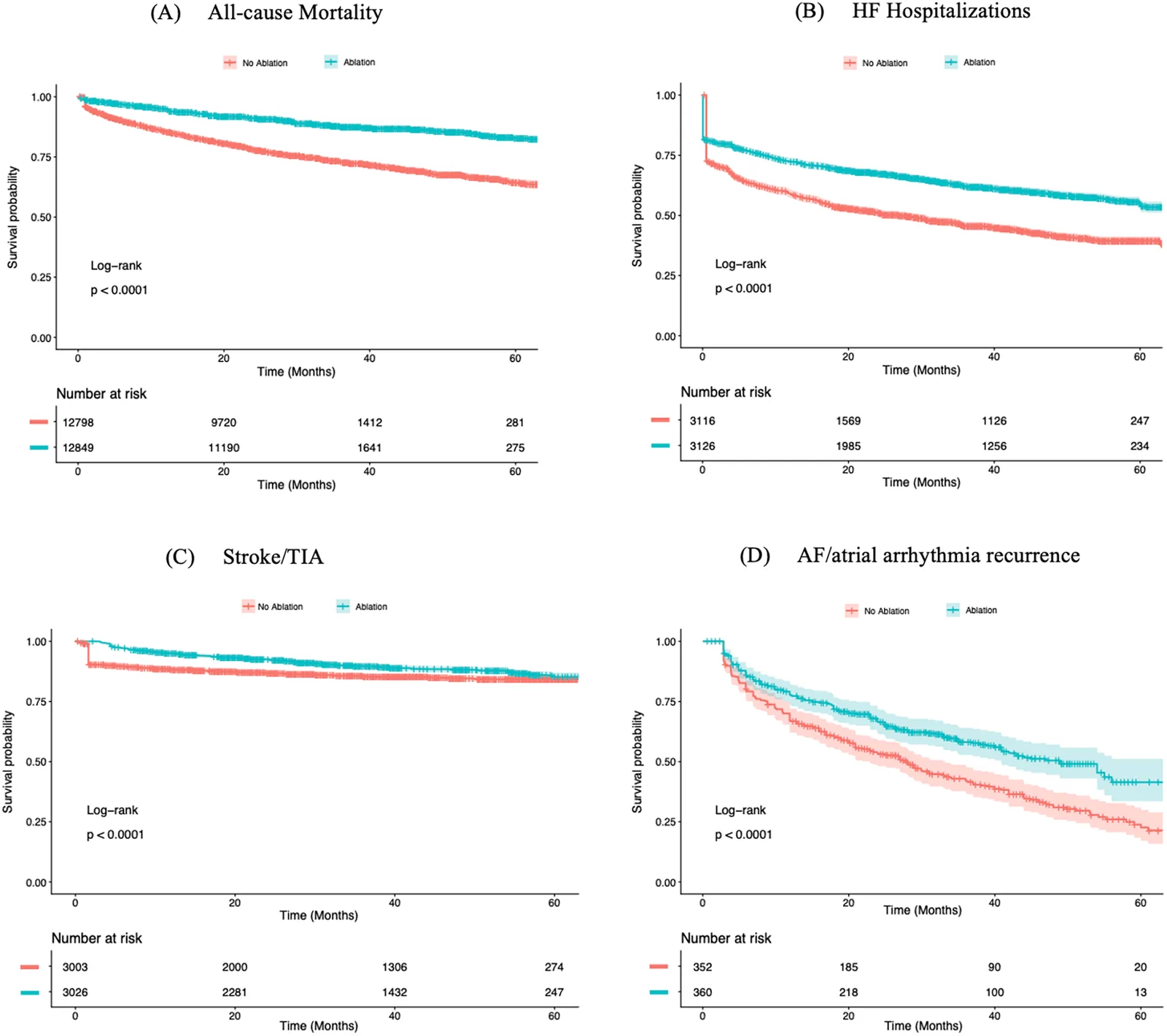
Kaplan–meier curves for freedom from **(A)** All-cause mortality, **(B)** HF hospitalizations, **(C)** stroke/TIA, and **(D)** AF/atrial arrhythmia recurrence, comparing catheter ablation versus medical therapy in patients with AF and HFpEF. Survival data were reconstructed from published KM curves using an individual patient data approach. Log-rank *p* values are shown.

In reconstructed time-to-event analyses of 712 participants, Kaplan–Meier estimates at 60 months demonstrated higher freedom from recurrence in the ablation group compared with control (41.4% [95% CI 33.6–51.2] vs. 22.6% [95% CI 17.1–29.9]). Frailty-adjusted Cox modeling confirmed a significant reduction in recurrence risk (HR: 0.651 [95% CI 0.524–0.808], *p* < 0.001) (Figure 6).

#### 3.3.3 All-Cause hospitalizations

Four studies reported this outcome (Supplementary Material S1, Supplementary Figure S6). The pooled hazard ratio that was reported for catheter ablation compared to medical therapy alone was 0.90 (95% CI: 0.81–1.00; *p* = 0.05), indicating a 10% reduction in the hospitalization rates of patients who underwent catheter ablation. The heterogeneity among the studies was low (I^2^ = 9%) and supports consistency of results across the studies.

#### 3.3.4 Changes in NT-proBNP

Data on change in NT-proBNP following catheter ablation vs. medical therapy or no ablation were available from two observational studies that reported sufficient information to calculate mean change and SD from baseline to follow-up Chieng et al, & Rattka et al., (11, 20).

Chieng et al. (11) reported NT-proBNP values at baseline and 6-month follow-up, from which mean change was calculated, showing a decrease in NT-proBNP in catheter ablation patients (−653 pg/mL, SD 656.9; *n* = 16), whereas the medical/no ablation group showed an increase (+90.4 pg/mL, SD 457.3; *n* = 15) (11).

Rattka et al. (20) reported baseline and follow-up NT-proBNP values over a mean follow-up of 35 ± 22 months.[20] In this study, NT-proBNP decreased in the ablation group (−1,377 pg/mL, SD 2,524.3; *n* = 43) and increased in the medical therapy group (+830 pg/mL, SD 3,597; *n* = 43).

Parkash et al. reported NT-proBNP using geometric mean, which could not be converted to a comparable metric and was therefore described narratively (18).

#### 3.3.5 Changes in left ventricular ejection fraction (LVEF)

Two studies provided data on changes in LVEF, though their reporting methods and follow-up times varied. When the data was standardized to show changes from baseline, both studies indicated that catheter ablation had a more favorable effect on LVEF than medical therapy, even though the total improvements were small.

Long et al. found that LVEF remained mostly stable in the ablation group (+0.33%) but dropped in the medical therapy group (−1.81%) over 36 months.^15^ Similarly, Rattka et al. noted a modest increase in LVEF with ablation (+2.6%) compared to a decrease in the medical therapy group (−2.63%) over nearly three years of follow-up.^20^ Because of the differences in how these studies were designed, the results were not combined into a single pool and should be viewed with caution.

Parkash et al. reported treatment effects as least-squares mean differences, which could not be converted to comparable change scores and were therefore described narratively (18).

## 4 Discussion

In this systematic review and meta-analysis of 13 studies, catheter ablation was compared with medical therapy in patients with AF and HFpEF. Our main findings were: (i) catheter ablation was associated with lower risks of all-cause mortality, HF hospitalization, stroke/TIA, and AF/atrial arrhythmia recurrence; (ii) all-cause hospitalization was numerically lower but only borderline significant; and (iii) exploratory outcomes suggested favourable trends in NT-proBNP and LVEF with ablation.

A recent study published based on the TOPCAT-Americas trial demonstrated that prevalent AF in HFpEF is independently associated with heart failure progression, heart failure hospitalisation, and pump failure death, while not conferring excess risk of sudden cardiac death. This suggests that AF primarily contributes to haemodynamic deterioration rather than arrhythmic mortality (23). In parallel, an analysis from the Framingham Heart Study showed that AF developing after HF onset is more strongly associated with all-cause mortality (24). Collectively, these observations provide a biological rationale for rhythm-control strategies aimed at reducing AF burden and preventing HF decompensation. The present analysis demonstrated significant reductions in all-cause mortality and heart failure hospitalisations with catheter ablation, consistent with prior evidence. Across most included studies, the direction of effect favoured catheter ablation, with heterogeneity largely reflecting differences in the magnitude rather than the direction of effect. However, Arora et al. (10), which had a relatively short follow-up duration, demonstrated a neutral association. This suggests that benefits of catheter ablation on hard clinical outcomes may require longer follow-up to become evident, a conclusion further supported by prespecified subgroup analyses of studies with longer follow-up. This interpretation was further supported by reconstructed individual patient–level analyses, which yielded concordant findings that reinforced the primary aggregate results.

Although catheter ablation was associated with a reduction in heart failure hospitalizations, the effect on all-cause hospitalizations was more modest. This finding is not unexpected, as all-cause hospitalization represents a broader and less specific endpoint influenced by comorbidity burden, healthcare utilisation, and non-cardiovascular admissions. As such, improvements in HF-related outcomes may not uniformly translate into large reductions in overall hospitalization rates, particularly in older HFpEF populations with multimorbidity.

Heart failure is a major modifier of thromboembolic risk in atrial fibrillation and is incorporated into CHA_2_DS_2_-VASc risk stratification scheme. In addition, AF promotes atrial structural and endothelial abnormalities that facilitate blood stasis and thrombogenesis, while concomitant heart failure, including HFpEF, further amplifies these haemodynamic disturbances, providing a mechanistic basis for increased thromboembolic risk and adverse clinical outcomes (25). The present analysis demonstrated a statistically significant association between catheter ablation and a reduced incidence of stroke or TIA in patients with atrial fibrillation and HFpEF, with low heterogeneity observed between studies. Catheter ablation was also associated with a significantly lower risk of atrial fibrillation or atrial arrhythmia recurrence compared to medical therapy alone. Although heterogeneity is low, the small number of analysable studies warrants caution in interpreting the results as evidence of association rather than causation.

A retrospective study reported that restoration of sinus rhythm following catheter ablation was associated with improvement in functional status, left atrial (LA) and left ventricular (LV) function, and plasma BNP levels in non-paroxysmal AF patients with HFpEF (26). Additional studies also support improvements in LV function in this population (27, 28). In the present review, continuous outcomes were limited and heterogeneous, with few studies reporting extractable data suitable for quantitative synthesis. Across available studies, catheter ablation was consistently associated with favourable changes in NT-proBNP compared with medical therapy (11, 20, 28). Collectively, these findings suggest that ablation may be favourably associated with haemodynamic stress and atrioventricular coupling in HFpEF. In contrast, changes in LVEF were modest across available studies. Long et.al and Rattka et.al demonstrated preservation rather than substantial improvement in systolic function, while Parkash et.al reported a sustained increase in LVEF favouring ablation over rate control (15, 18, 20). Taken together, these findings suggest that the clinical benefits associated with catheter ablation in HFpEF may not be mediated primarily through improvements in systolic function but rather through preservation of ventricular performance and favourable structural or diastolic remodeling.

Several impactful studies have established the significance of ablation as a treatment strategy for AF in HFrEF cohort (29, 30), and current guidelines have incorporated this evidence (5). While HFpEF is the most common HF phenotype in the AF group, evaluating for an optimal treatment strategy can benefit this large population. To corroborate this evidence gap, many studies have been published in the past few years, but large HFpEF-focused RCTs are still lacking. Ongoing large-scale trials like CABA-HFPEF, TAP-CHF, and STABLE-SR-IV, are expected to provide more robust evidence regarding the role of catheter ablation in HFpEF.

## 5 Strengths and limitations

Strengths of this study include the focus on HFpEF-specific populations, efforts to minimize phenotypic overlap, inclusion of recently published studies not included in prior meta-analyses, and evaluation of both clinical outcomes and mechanistic endpoints. Time-to-event outcomes were pooled using hazard ratios when available, with reconstructed IPD Kaplan–Meier analyses performed to validate survival estimates. Sensitivity analyses were conducted to explore heterogeneity and assess the robustness of findings.

This analysis presents several limitations. First, although propensity score matching and multivariable adjustment were employed in many of the included observational studies, residual confounding and indication bias cannot be completely excluded. Patients selected for catheter ablation may have differed from medically managed patients in ways not fully captured by measured covariates, including frailty, symptom burden, functional status, comorbidity profile and suitability for invasive intervention, potentially resulting in overestimation of treatment effects. Second, definitions of HFpEF varied across studies, which may have contributed to heterogeneity despite efforts to prioritize clearly defined populations. Furthermore, HFpEF represents a heterogeneous syndrome comprising multiple clinical phenotypes with distinct pathophysiological mechanisms and prognostic profiles. The included studies did not consistently report phenotype-specific data, precluding subgroup analyses according to HFpEF phenotype and limiting assessment of whether catheter ablation benefits differ across HFpEF subgroups. Follow-up duration also varied across studies, potentially limiting the evaluation of long-term outcomes such as mortality and heart failure hospitalizations in shorter cohorts. For stroke/TIA, the primary analysis was performed using risk ratios because time-to-event estimates were not consistently reported across all studies. Unlike HRs, RRs do not account for differences in follow-up duration and therefore are not directly equivalent to time-to-event estimates. Accordingly, the stroke/TIA findings should be interpreted with this methodological consideration in mind, although an HR-based sensitivity analysis yielded consistent results. Although attempts were made to minimize population overlap, inclusion of registry and administrative database studies means that complete exclusion of overlapping participants cannot be guaranteed. Finally, IPD-KM reconstruction was feasible only for studies reporting analyzable Kaplan–Meier curves and included smaller populations than the aggregate analyses, potentially limiting generalizability. Finally, one study with a lower ejection fraction threshold (≥45%) was included only in narrative synthesis and not in pooled analyses (18).

## 6 Conclusion

In this meta-analysis of patients with AF and HFpEF, catheter ablation was associated with significant reductions in all-cause mortality, heart failure hospitalizations, stroke, and AF/atrial arrhythmia recurrence compared with medical therapy. Ablation also showed a favorable trend toward reduced all-cause hospitalizations, although this did not reach statistical significance. Individual studies reported reductions in NT-proBNP and modest improvements in LVEF, but these outcomes were not consistently reported or pooled.

Overall, these findings suggest that catheter ablation may improve outcomes in patients with HFpEF. However, heterogeneity in HFpEF definitions, study designs, and follow-up durations warrants cautious interpretation. Large, HFpEF-focused randomized trials are needed to confirm these findings and identify patients most likely to benefit.

## Data Availability

The original contributions presented in the study are included in the article/Supplementary Material, further inquiries can be directed to the corresponding author/s.
