# Supplementary material for: Prognostic impact of catheter ablation in atrial fibrillation with heart failure with preserved ejection fraction: an updated systematic review and reconstructed individual patient data meta-analysis
Source: Front Cardiovasc Med. 2026 Aug 19;13:1883350. doi: 10.3389/fcvm.2026.1883350 (PMC13533950; doi:10.3389/fcvm.2026.1883350)
Supplement: Supplementary file 1 [file Datasheet1.pdf]

Supplementary Material 1

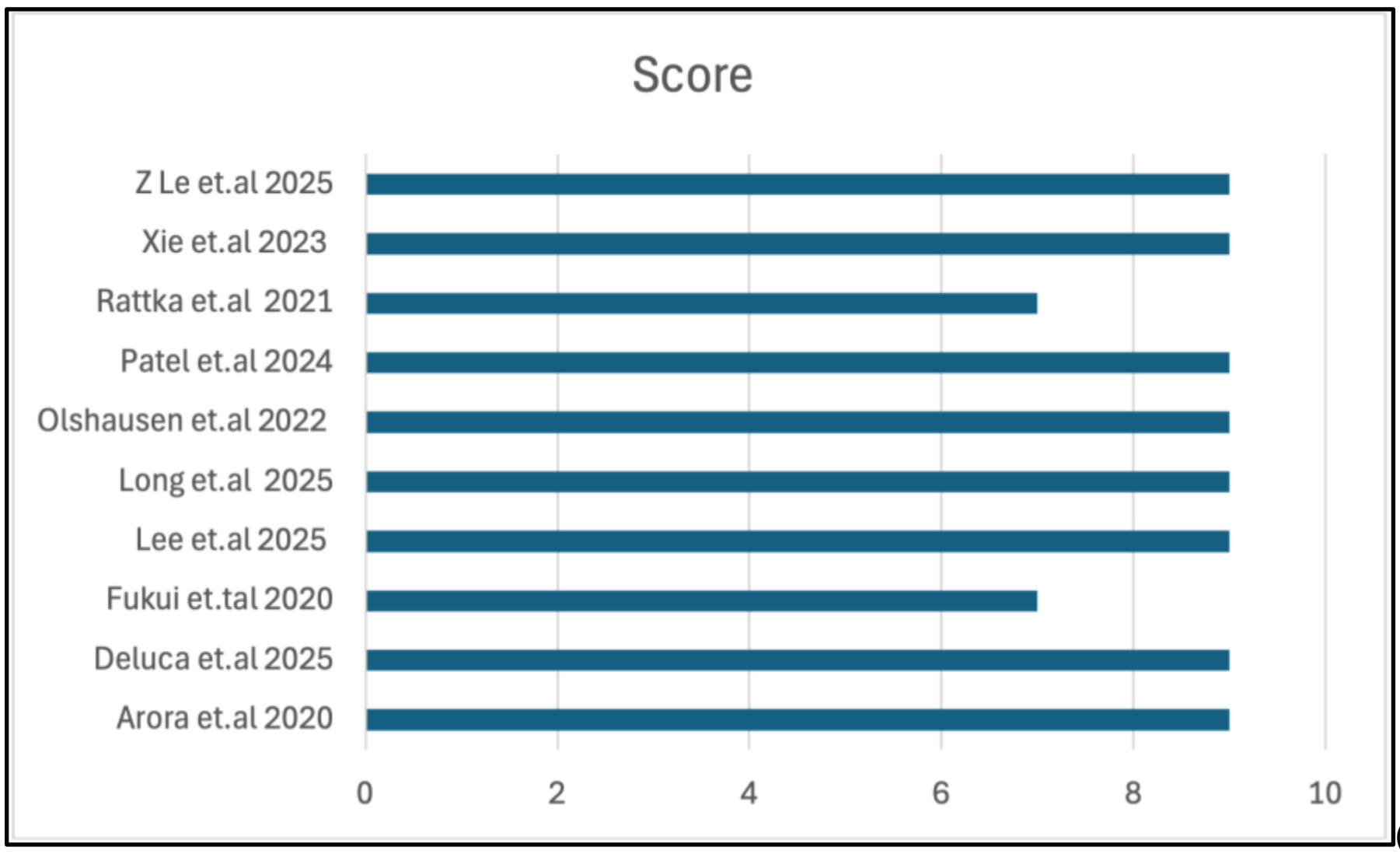

(i)

S.No	Study ID	SELECTION	COMPARABILITY	OUTCOME	OVERALL SCORE
1	Arora et.al 2020	4	2	3	9
2	Deluca et.al 2025	4	2	3	9
3	Fukui et.al 2020	3	1	3	7
4	Lee et.al 2025	4	2	3	9
5	Long et.al 2025	4	2	3	9
6	Olshausen et.al 2022	4	2	3	9
7	Patel et.al 2024	4	2	3	9
8	Rattka et.al 2021	3	1	3	7
9	Xie et.al 2023	4	2	3	9
10	Z Le et.al 2025	4	2	3	9

(ii)

Supplementary figure 1: Newcastle Ottawa Scale(NOS) assessment for observational studies. (i) Graphical presentation of NOS scores (ii) Domain-level NOS Table. NOS scores range from 0 to 9, with higher scores indicating methodological quality.

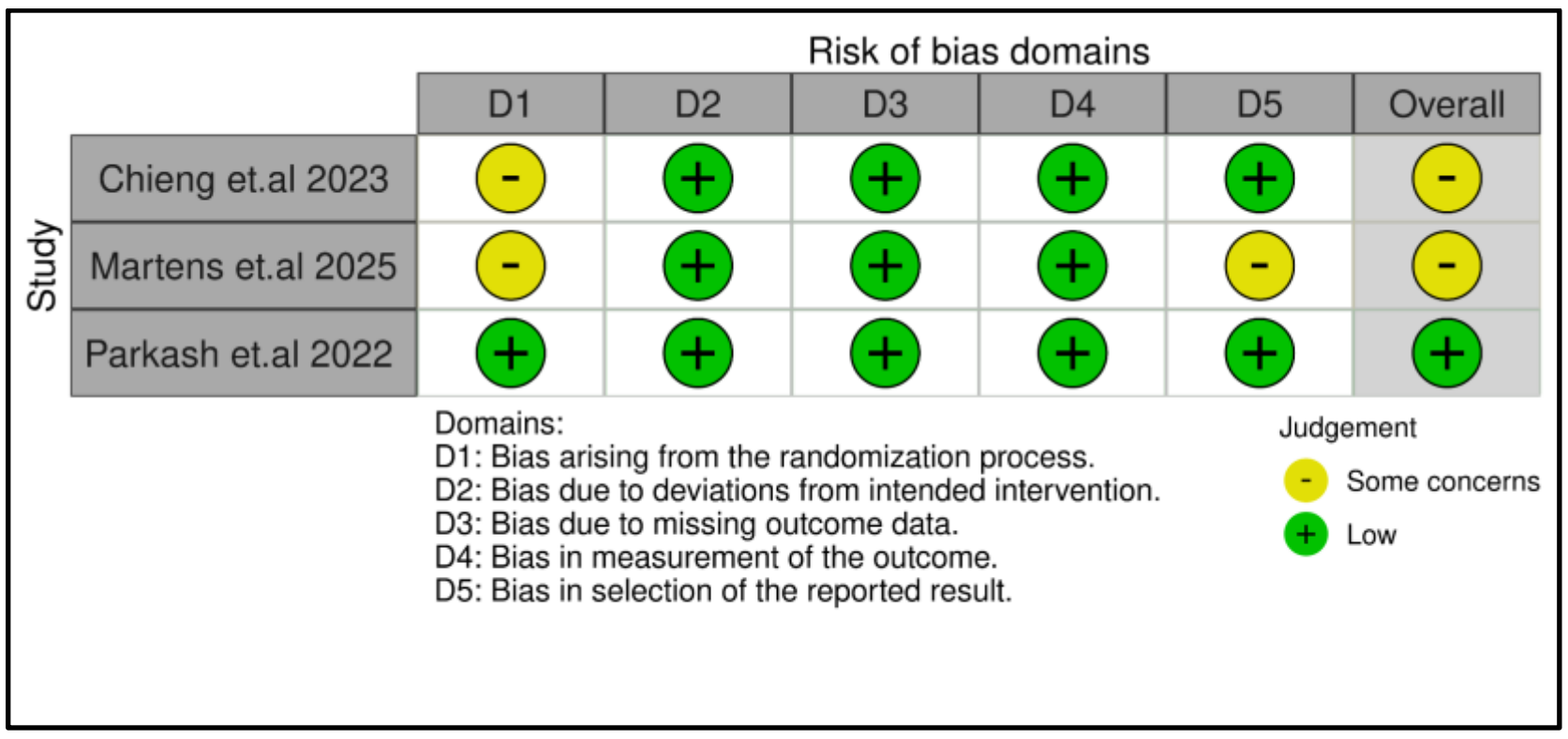

Supplementary figure 2: Risk of bias assessment for randomized controlled trials using Cochrane Risk of Bias 2 (ROB2) tool.

Study or Subgroup	log[HR]	SE	Weight	Hazard ratio IV, Random, 95% CI
**1.10.1 Longer follow-up duration (median > 2 years)**				
Deluca etal 2024	-0.841647	0.093536	13.6%	0.43 [0.36 , 0.52]
Lee etal 2025	-1.07881	0.13421	12.8%	0.34 [0.26 , 0.44]
Long etal 2025	-0.71335	0.327863	8.2%	0.49 [0.26 , 0.93]
Olashausen etal 2022	-0.400478	0.326775	8.2%	0.67 [0.35 , 1.27]
Xie etal 2023	-0.562119	0.404622	6.7%	0.57 [0.26 , 1.26]
Z.Le etal 2025	-0.616186	0.266618	9.6%	0.54 [0.32 , 0.91]
**Subtotal (Wald[a])**			**59.3%**	**0.44 [0.36 , 0.53]**
Test for overall effect: Z = 8.26 (P < 0.00001)				
Heterogeneity: Tau² (REML[b]) = 0.02; Chi² = 6.25, df = 5 (P = 0.28); I² = 30%				
**1.10.2 Shorter follow-up duration (median ≤ 2 years)**				
Arora etal 2020	0.076961	0.138278	12.7%	1.08 [0.82 , 1.42]
Martens etal 2025	-0.198451	0.089507	13.7%	0.82 [0.69 , 0.98]
Patel etal 2024	-0.941609	0.039325	14.3%	0.39 [0.36 , 0.42]
**Subtotal (Wald[a])**			**40.7%**	**0.69 [0.38 , 1.27]**
Test for overall effect: Z = 1.19 (P = 0.23)				
Heterogeneity: Tau² (REML[b]) = 0.27; Chi² = 97.31, df = 2 (P < 0.00001); I² = 98%				
**Total (Wald[a])**			**100.0%**	**0.55 [0.42 , 0.73]**

Test for overall effect: Z = 4.15 (P < 0.0001)
Test for subgroup differences: Chi² = 2.03, df = 1 (P = 0.15), I² = 50.6%
Heterogeneity: Tau² (REML[b]) = 0.14; Chi² = 104.95, df = 8 (P < 0.00001); I² = 91%

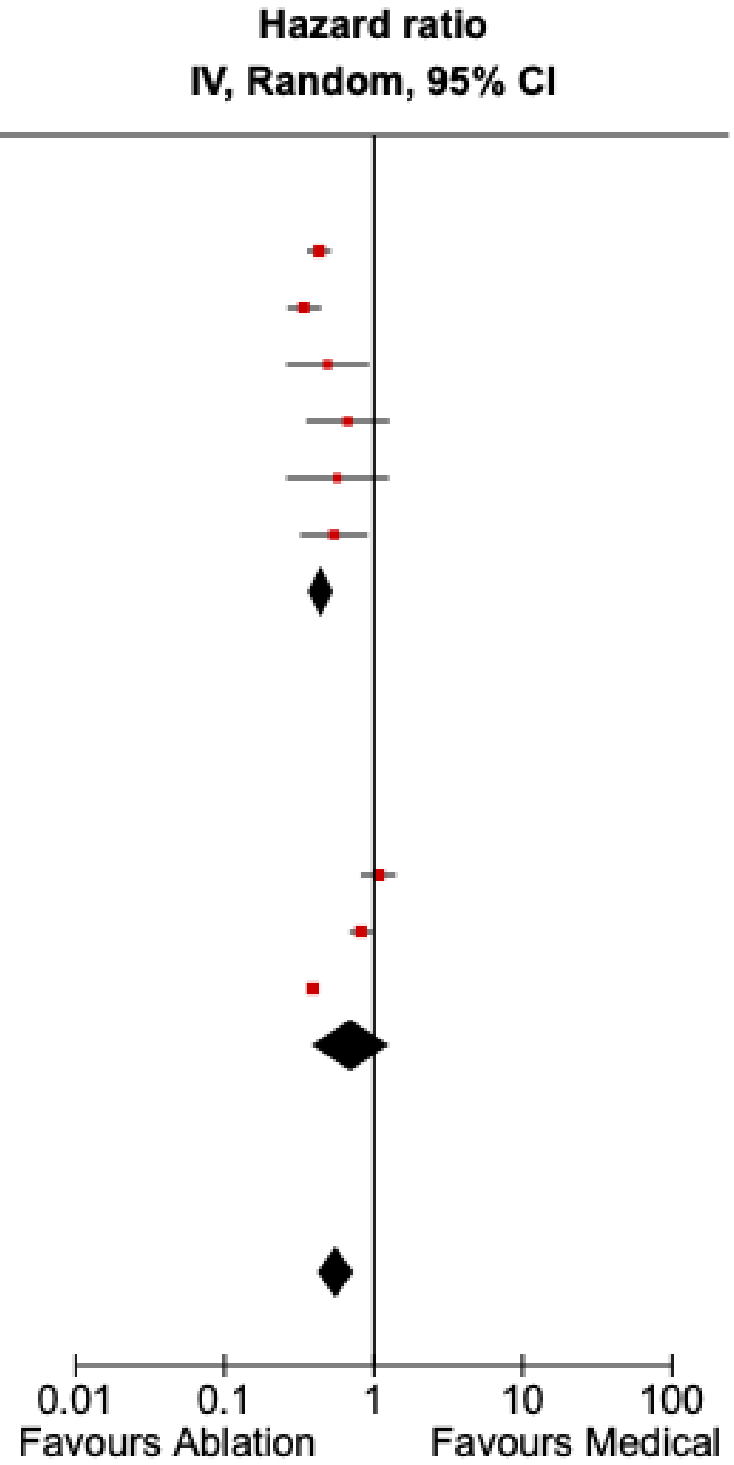

Footnotes
[a]CI calculated by Wald-type method.
[b]Tau² calculated by Restricted Maximum-Likelihood method.

Supplementary figure 3: Pre-specified subgroup analysis of all-cause mortality by follow-up duration. Hazard ratios with 95% confidence intervals are shown. CI = Confidence interval; HR= hazard ratio; IV= inverse variance.

Study or Subgroup	log[HR]	SE	Weight	Hazard ratio IV, Random, 95% CI
**1.9.1 Longer follow-up duration (median >2 years)**				
Deluca etal 2024 1[a]	-0.449417	0.076043	16.6%	0.64 [0.55 , 0.74]
Deluca etal 2024 2[b]	-0.438505	0.181302	12.9%	0.64 [0.45 , 0.92]
Lee etal 2025	-0.248461	0.080893	16.5%	0.78 [0.67 , 0.91]
Rattka etal 2021	-1.203973	0.41831	6.0%	0.30 [0.13 , 0.68]
Xie etal 2023	-0.653926	0.218286	11.5%	0.52 [0.34 , 0.80]
**Subtotal (Wald[c])**			**63.6%**	**0.65 [0.54 , 0.77]**
Test for overall effect: Z = 4.95 (P < 0.00001)				
Heterogeneity: Tau^2 (REML[d]) = 0.02; Chi^2 = 8.99, df = 4 (P = 0.06); I^2 = 50%				
**1.9.2 Shorter follow-up duration (median ≤ 2 years)**				
Arora etal 2020	0.067659	0.115035	15.4%	1.07 [0.85 , 1.34]
Fukui etal 2020	-1.714798	0.565208	3.9%	0.18 [0.06 , 0.54]
Patel etal 2024	-0.150823	0.054318	17.1%	0.86 [0.77 , 0.96]
**Subtotal (Wald[c])**			**36.4%**	**0.63 [0.25 , 1.61]**
Test for overall effect: Z = 0.96 (P = 0.34)				
Heterogeneity: Tau^2 (REML[d]) = 0.60; Chi^2 = 10.94, df = 2 (P = 0.004); I^2 = 97%				
**Total (Wald[c])**			**100.0%**	**0.67 [0.52 , 0.86]**

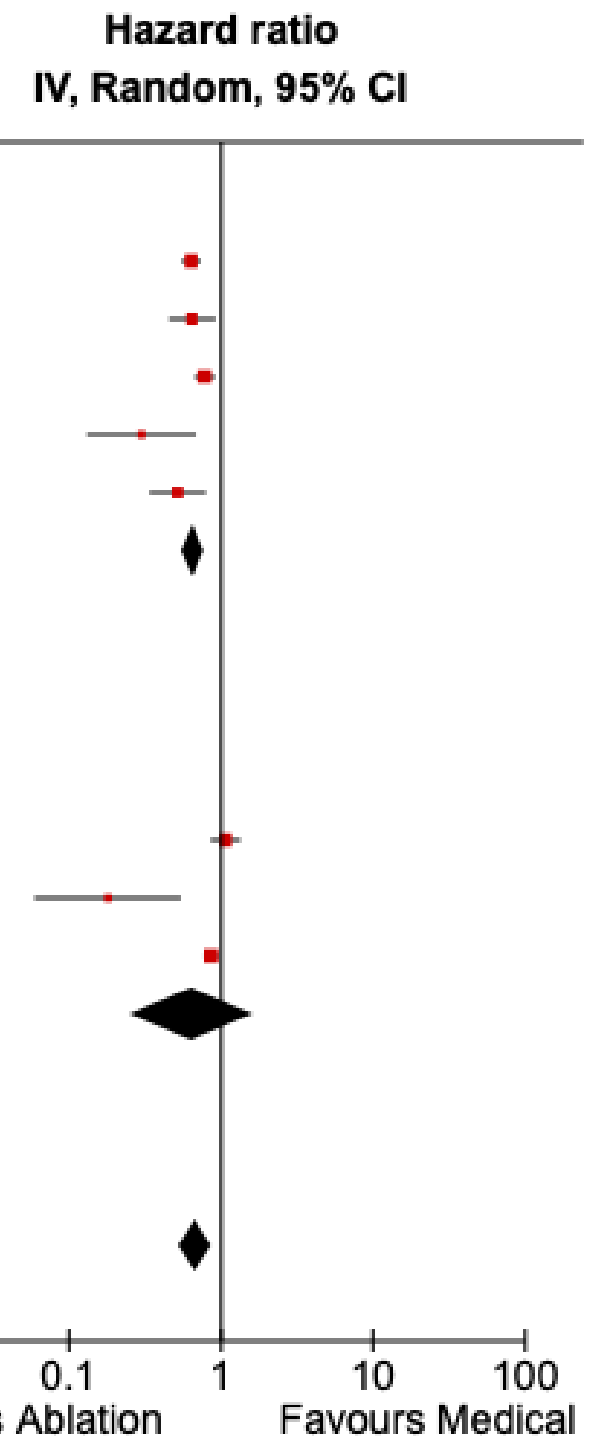

Test for overall effect: Z = 3.20 (P = 0.001)
Test for subgroup differences: Chi^2 = 0.00, df = 1 (P = 0.96), I^2 = 0%
Heterogeneity: Tau^2 (REML[d]) = 0.09; Chi^2 = 34.26, df = 7 (P < 0.0001); I^2 = 88%

Footnotes
[a]Acute HFpEF Hospitalisations
[b]Acute HFrEF Hospitalisations
[c]CI calculated by Wald-type method.
[d]Tau^2 calculated by Restricted Maximum-Likelihood method.

Supplementary figure 4: Pre-specified subgroup analysis of HF hospitalization by follow-up duration. Hazard ratios with 95% confidence intervals are shown. CI = Confidence interval; HR= hazard ratio; IV= inverse variance.

Study or Subgroup	log[HR]	SE	Weight	Hazard ratio IV, Random, 95% CI
Deluca etal 2024	-0.067209	0.130033	25.0%	0.93 [0.72 , 1.21]
Lee Etal 2025	-0.328504	0.122129	26.5%	0.72 [0.57 , 0.91]
Olshausen et al 2022	-0.261365	0.589522	2.3%	0.77 [0.24 , 2.45]
Patel Etal 2024	-0.415515	0.054318	42.3%	0.66 [0.59 , 0.73]
Xie Etal 2023	-0.371064	0.454035	3.8%	0.69 [0.28 , 1.68]
**Total (Wald[a])**			**100.0%**	**0.74 [0.62 , 0.89]**

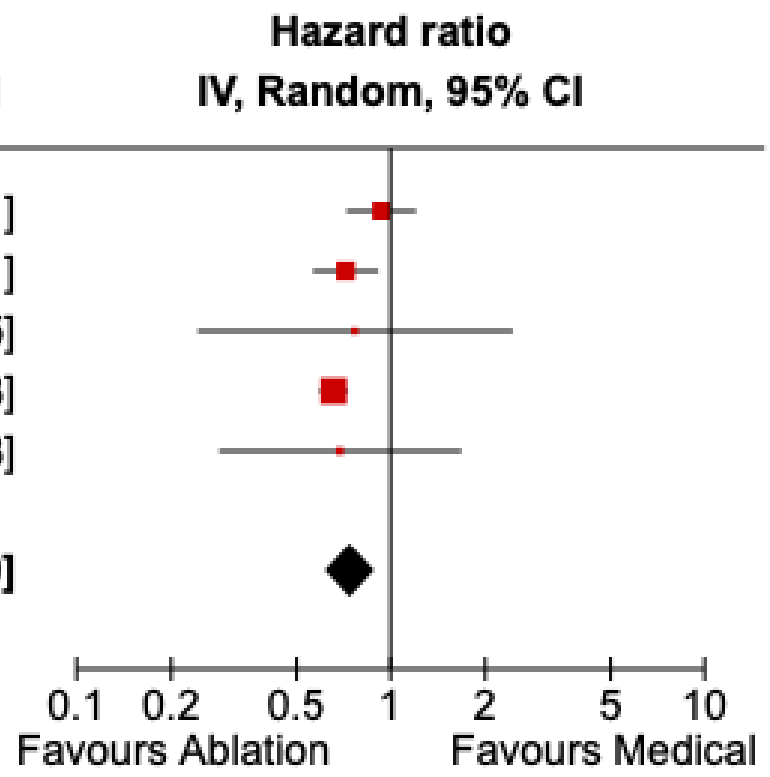

Test for overall effect: Z = 3.25 (P = 0.001)

Heterogeneity: Tau² (REML[b]) = 0.02; Chi² = 6.21, df = 4 (P = 0.18); I² = 48%

Footnotes

[a]CI calculated by Wald-type method.

[b]Tau² calculated by Restricted Maximum-Likelihood method.

Supplementary figure 5: Sensitivity analysis restricted to studies reporting to Hazard Ratios for stroke/TIA. Hazard ratios with 95% confidence intervals are shown. CI = Confidence interval; HR= hazard ratio; IV=inverse variance.

Study or Subgroup	log[HR]	SE	Weight	Hazard ratio IV, Random, 95% CI
Arora etal 2020	-0.061875	0.051465	70.1%	0.94 [0.85 , 1.04]
Olashausen etal 2022	-0.162519	0.181043	8.8%	0.85 [0.60 , 1.21]
Rattka etal 2021	-0.579818	0.272413	4.0%	0.56 [0.33 , 0.96]
Xie etal 2023	-0.150823	0.126479	17.1%	0.86 [0.67 , 1.10]
**Total (Wald[a])**			**100.0%**	**0.90 [0.81 , 1.00]**

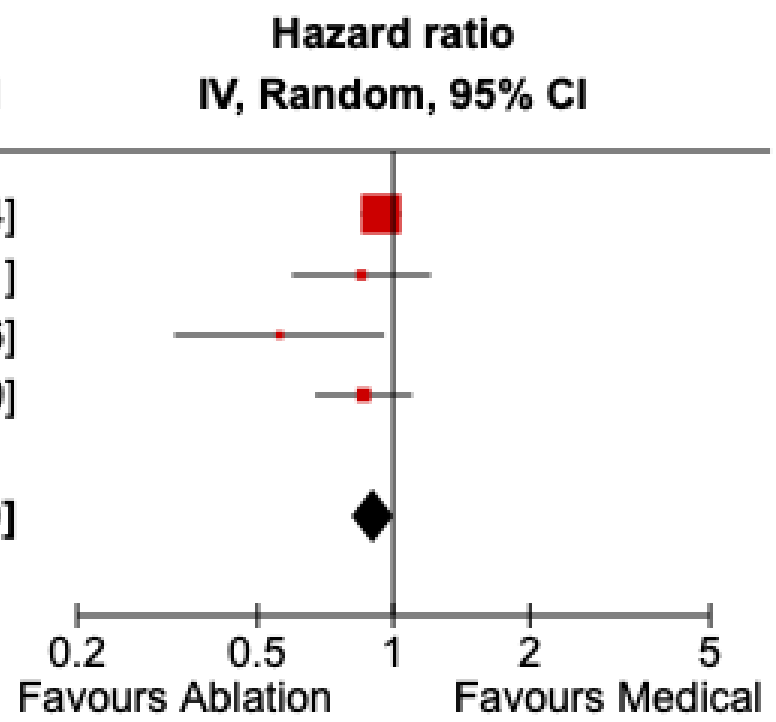

Test for overall effect: Z = 1.94 (P = 0.05)

Heterogeneity: Tau² (REML[b]) = 0.00; Chi² = 3.92, df = 3 (P = 0.27); I² = 9%

Footnotes

[a]CI calculated by Wald-type method.

[b]Tau² calculated by Restricted Maximum-Likelihood method.

Supplementary figure 6: Forest plot showing All-cause hospitalizations comparing catheter ablation vs medical therapy. Hazard ratios with 95% confidence intervals are shown. CI = Confidence interval; HR= hazard ratio; IV= inverse variance.

Full Search Strategy for Screening across databases:

PUBMED
"atrial fibrillation"[Title] AND ("ablation"[Title] AND "catheter"[Title/Abstract]) AND ("HFpEF"[Title] OR "Heart failure"[Title])
Results : 324, Date : 8 Dec 2025

EMBASE
'atrial fibrillation':ti AND 'ablation':ti AND 'catheter':ab,ti AND ('hfpef':ti OR 'heart failure':ti)
Results : 529, Date : 8 Dec 2025

SCOPUS
(TITLE ("atrial fibrillation") AND TITLE ("ablation") AND TITLE-ABS ("catheter") AND TITLE ("HFpEF" OR "Heart failure"))
Results : 307, Date : 8 Dec 2025
