# Supplementary material for: Prognostic impact of catheter ablation in atrial fibrillation with heart failure with preserved ejection fraction: an updated systematic review and reconstructed individual patient data meta-analysis
Source: Front Cardiovasc Med. 2026 Aug 19;13:1883350. doi: 10.3389/fcvm.2026.1883350 (PMC13533950; doi:10.3389/fcvm.2026.1883350)
Supplement: Supplementary file 2 [file Datasheet2.docx]

**Supplementary Material 2**

| S. No | Study (Year) | Number of patients | | Age (Mean ± SD) | | Male | | LVEF (Mean ± SD) | | CHAD-VASc- Score (Mean ± SD) | | Persistent AF | | Paroxysmal AF | |
| --- | --- | --- | --- | --- | --- | --- | --- | --- | --- | --- | --- | --- | --- | --- | --- |
|  |  | CA | Med | CA | Med | CA | Med | CA | Med | CA | Med | CA | Med | CA | Med |
| 1 | Arora et al., 2020(10) | 1053 | 16848 | 76 ±10 | 74±12 | 377 (35.8%) | 5702 (36.1%) | - | - | - | - | 256 | 3538 | 484 | 7483 |
| 2 | Chieng et al., 2023(11) | 16 | 15 | 65.5±7.6 | 66.7±7.9 | 8 (50%) | 7 (46.7%) | 60.4±5.3 | 58.5±4.6 | 3±2 | 4±2 | 13 | 12 | 3 | 2 |
| 3 | DeLuca et al., 2025(12) | 1816 | 1816 | 63.7±8.2 | 63.4±8.9 | 1030(56.7%) | 1025 (56.4%) | - | - | - | - | 1217 | 1236 | - | - |
| 4 | Fukui et al., 2020(13) | 35 | 50 | 70±8 | 71±13 | 23(65.7%) | 32(64%) | 62±8 | 61±9 | - | - | - | - | - | - |
| 5 | Lee et al., 2025(14) | 1152 | 1152 | 68.3±9.2 | 68.1±13.2 | 605(52.5%) | 614(53.3%) | - | - | - | - | - | - | 863 | 858 |
| 6 | Long et al., 2025(15) | 187 | 187 | 65.81±8.18 | 66.1±7.61 | 99(52.9%) | 102(54.5%) | 56.49±2.5 | 56.85±3.4 | 3.68±1.33 | 3.18±1.17 | 83 | 76 | 87 | 95 |
| 7 | Martens et al., 2025(16) | 488 | 486 | - | - | - | - | - | - | - | - | - | - | - | - |
| 8 | Olshausen et al.,  2022(17) | 72 | 151 | - | - | - | - | - | - | - | - | - | - | - | - |
| 9 | Parkash et al., 2022(18) | 214 | 197 | 65.9±8.6 | 67.5±8 | 157(73.4%) | 148(75.1%) | 54.6±7.3 | 55.9±6.7 | - | - | 188 | 186 | 19 | 11 |
| 10 | Patel et al., 2024(19) | 9440 | 9440 | 68±9.9 | 67.2±12.6 | 5201 (55.1%) | 5428 (57.5%) | - | - | - | - | 5617 | 5494 | 8052 | 8184 |
| 11 | Rattka et al., 2021(20) | 43 | 43 | 73±7 | 74±7 | - | - | 62.8±10 | 65.4±10.4 | 4±1.5 | 4±1.5 | - | - | 26 | 22 |
| 12 | Xie et al., 2023(21) | 293 | 293 | 68±7.5 | 67.3±8.1 | - | - | 62.7±5.2 | 62.7±5.2 | 3±1.49 | 3±1.49 | 186 | 181 | 107 | 112 |
| 13 | Z. Le et al., 2025(22) | 604 | 604 | 62.9±9.9 | 62.3±10.8 | 363(60.1%) | 382(63.2%) | 54.7±13 | 53.9±14 | 3±1.5 | 3±1.5 | 323 | 322 | - | - |

**Online Resource Table 1:** Detailed baseline demographics of included studies.

Abbreviations:

AF = Atrial fibrillation; SD = standard deviation; LVEF = Left Ventricular Ejection Fraction; CHAD-VASc score= clinical prediction score used to estimate the risk of stroke in patients with atrial fibrillation; CA = catheter ablation; Med = Medical therapy.
